# Pesticides in ambient air, influenced by surrounding land use and weather, pose a potential threat to biodiversity and humans

**DOI:** 10.1016/j.scitotenv.2022.156012

**Published:** 2022-05-18

**Authors:** Johann G. Zaller, Maren Kruse-Plaß, Ulrich Schlechtriemen, Edith Gruber, Maria Peer, Imran Nadeem, Herbert Formayer, Hans-Peter Hutter, Lukas Landler

**Affiliations:** a University of Natural Resources and Life Sciences Vienna (BOKU), Department of Integrative Biology and Biodiversity Research, Institute of Zoology, Gregor Mendel Straße 33, 1180 Vienna, Austria; b TIEM Integrated Environmental Monitoring, 95615 Marktredwitz, Germany; c TIEM Integrated Environmental Monitoring, Hohenzollernstr. 20, 44135 Dortmund, Germany; d University of Natural Resources and Life Sciences Vienna (BOKU), Department of Water, Atmosphere and Environment, Institute of Meteorology and Climatology, Peter-Jordan Straße 82, 1180 Vienna, Austria; e Department of Environmental Health, Center for Public Health, Medical University Vienna, Kinderspitalgasse 15, 1090 Vienna, Austria

**Keywords:** Agrochemicals, Off-target area, Pesticide drift, Exposure, Agriculture, Human toxicology, Air pollution, Toxic loads, Environmental risk assessment

## Abstract

Little is known about (i) how numbers and concentrations of airborne pesticide residues are influenced by land use, interactions with meteorological parameters, or by substance-specific chemo-physical properties, and (ii) what potential toxicological hazards this could pose to non-target organisms including humans. We installed passive air samplers (polyurethane PUF and polyester PEF filter matrices) in 15 regions with different land uses in eastern Austria for up to 8 months. Samples were analyzed for 566 substances by gas-chromatography/mass-spectrometry. We analyzed relationships between frequency and concentrations of pesticides, land use, meteorological parameters, substance properties, and season. We found totally 67 pesticide active ingredients (24 herbicides, 30 fungicides, 13 insecticides) with 10–53 pesticides per site. Herbicides metolachlor, pendimethalin, prosulfocarb, terbuthylazine, and the fungicide HCB were found in all PUF samplers, and glyphosate in all PEF samplers; chlorpyrifos-ethyl was the most abundant insecticide found in 93% of the samplers. Highest concentrations showed the herbicide prosulfocarb (725 ± 1218 ng sample^−1^), the fungicide folpet (412 ± 465 ng sample^−1^), and the insecticide chlorpyrifos-ethyl (110 ± 98 ng sample^−1^). Pesticide numbers and concentrations increased with increasing proportions of arable land in the surroundings. However, pesticides were also found in two National Parks (10 and 33 pesticides) or a city center (17 pesticides). Pesticide numbers and concentrations changed between seasons and correlated with land use, temperature, radiation, and wind, but were unaffected by substance volatility. Potential ecotoxicological exposure of mammals, birds, earthworms, fish, and honeybees increased with increasing pesticide numbers and concentrations. Human toxicity potential of detected pesticides was high, with averaged 54% being acutely toxic, 39% reproduction toxic, 24% cancerogenic, and 10% endocrine disrupting. This widespread pesticide air pollution indicates that current environmental risk assessments, field application techniques, protective measures, and regulations are inadequate to protect the environment and humans from potentially harmful exposure.

## Graphical abstract

**Graphical Abstract:**
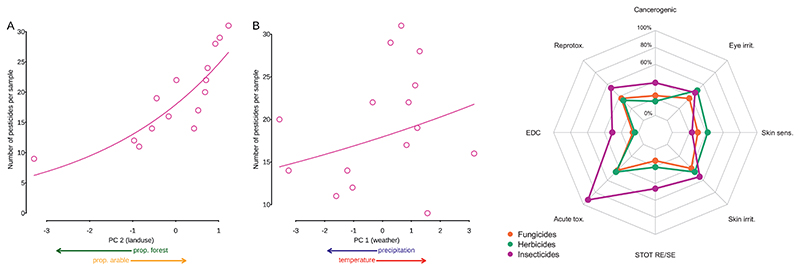

## 1 Introduction

Global pesticide use has increased dramatically in recent decades, resulting in increasing exposure of all organisms and the environment (Tang et al., 2021; Zaller, 2020). Areas near pesticide-intensive agriculture are particularly affected, as reported for the USA (Córdoba Gamboa et al., 2020; Fenske et al., 2002), China (Liu et al., 2014), Costa Rica (van Wendel de Joode et al., 2012), Japan (Kawahara et al., 2005), South-Africa (Dalvie et al., 2014), northern Italy (Linhart et al., 2019; Linhart et al., 2021), Germany (Brühl et al., 2021a; Kruse-Plaß et al., 2021), and many other countries. Various media can be analyzed to monitor pesticide contamination and it is clear that aerial dispersal is an important pathway for the spread of pesticides in the environment (Bidleman and Leone, 2004; Gibbs et al., 2017; Lu et al., 2000). However, little is known about how the number and concentration of pesticide residues in the air are affected by land use, meteorological parameters, or compound-specific chemical-physical properties, and what toxicological hazards this might pose to non-target organisms, including humans.

In general, pesticides in air are the result of application-related drift from (i) pesticide droplets that evaporate before they hit the target and travel with fine spray particles, (ii) post application drift of vapors from the target area, and (iii) drift from wind or wind-blown soil particles (Mueller, 2015). Off-target pesticide contamination in public places has been shown to correlate with distance from their application fields and the proportion of pesticide treated fields in the landscape (Linhart et al., 2019). In addition, inherent chemical properties may be important for drift (Schampheleire et al., 2009). Some chemicals are inherently volatile and can evaporate depending on the vapor pressure of the substance in the atmosphere (the lower, the more volatile), temperature (the higher, the more volatile) and relative humidity of the air (the higher humidity, the lower the evaporation) (Bish et al., 2021). Precipitation can reduce pesticide vaporization but increase pesticide leaching (Zaller et al., 2021). Therefore, it can be hypothesized that lower ambient temperatures and a low-volatility of a pesticide would reduce pesticide contamination in air samples.

Little research has been conducted on interactions between land use, meteorology and substance properties and their toxicological consequences of airborne pesticide exposure of wildlife or humans. Using pesticide sales data, total potential toxic loads for honeybees (expressed as total LD_50_ doses applied) increased even though pesticide use substantially decreased over the last 25 years in the UK (Goulson et al., 2018). Potential toxic exposure of aquatic insects and pollinators to insecticides has also increased over the past 26 years in the USA as the classes of chemicals used have shifted, even as the amounts applied have decreased (Schulz et al., 2021). Depending on the pesticides considered, a decrease in pesticide use may also lead to lower toxic loads, as has been demonstrated for farmland bird species in UK (Tassin de Montaigu and Goulson, 2020). Pesticide contamination is not limited to agricultural land, but may also pose a toxicological threat to crops cultivated near application sites, non-target plants, livestock, sensitive nature conservation areas (Brühl et al., 2021a), fish and wildlife (Tang et al., 2021). Pesticide drift can also expose farm workers, bystanders and contaminate public places (Linhart et al., 2019; Linhart et al., 2021; Paglia et al., 2021; Sapcanin et al., 2016).

We conducted this study to: 1) assess environmental exposure and seasonal distribution patterns of pesticides via passive air sampling at 15 sites that differed in their surrounding land use; 2) investigate the influence of land use, meteorological parameters and specific chemical properties of pesticides on local contamination levels; and 3) evaluate potential toxicological hazards of these exposures of various non-target organisms and humans. The study was conducted in an agricultural landscape in eastern Austria, but also included sites in two national parks and a city center. We distributed samplers at high densities to maximize spatial resolution while studying a relatively small region. This strategy allowed us to analyze the detailed effects of landscape, weather, and season on pesticide levels at minimal local differences in pesticide-independent conditions.

We hypothesized that: (i) pesticide exposure correlates with spatial and temporal use in agricultural fields in the surroundings, (ii) high volatility of pesticides, higher temperatures, less rainfall, and low wind speeds promote pesticide occurrence in air samples; and (iii) higher pesticide levels lead to higher toxic loads of non-target organisms including humans. To the best of our knowledge, the present study is novel because it interactively examines multiple factors affecting pesticide exposure and its potential risk to nontarget organisms, including humans.

## 2 Materials and methods

### 2.1 Sampling locations

**Fig. 1:**
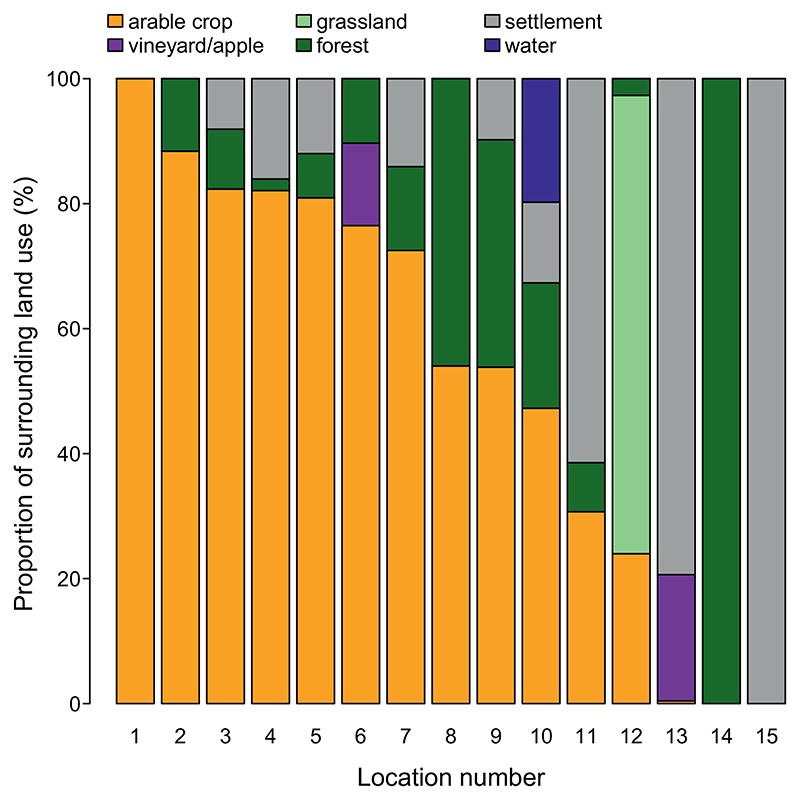
Proportion of land use categories surrounding air sampler locations within a 1 km radius based on the Austrian land use map and orthophotos.

We selected 15 sampling sites in a cultural landscape in eastern Austria to represent a land use gradient from 0% to 100% agricultural use within a 1 km radius. Surrounding land use included arable crops, managed grasslands, vineyards, or apple orchards. Additionally, one site was located in a city center 100% surrounded by settlements, and two sites located within two different National Parks of which one was surrounded by 100% forest and the other one by 73% grassland (Fig. 1). In order to protect privacy of the landowners, the exact location of the selected sites cannot be given here.

Field mapping was carried out in July 2015. As a reference we used the official Austrian land utilization map from 2012 (Integriertes Verwaltungsund Kontrollsystem, INVEKOS).

Land use types were mapped within a 1 km radius of the sampler site based on publicly available CORINE Land Cover database (UBA Wien and EEA, 2018) and EUNIS Habitat Classification (Davies et al., 2004). The most recent land cover database available was from 2018, while pesticide sampling was in 2020. In addition, freely available orthophotos from 2020 with an accuracy of 29 cm were used to adjust for potential land use changes (Geoland, 2020). Accordingly, the following land use types were distinguished: arable crops, vineyards and apple orchards, grassland, forest, settlements, and water bodies. Mapping and analysis were performed using ArcGis 10.2.195, QGis 2.8.1, FRAGSTATS 4.296 and CHLOE201297.

While we do not have data on the actual application of pesticides to agricultural fields during the study period, the land use categories indirectly provide information on the seasonal patterns of pesticide application. Assuming that fields were managed according to good agricultural practices, this included pesticide application, and we should be able to detect it in the air. For example, arable crops consisting mainly of cereals are sprayed until harvest in June/July, while vineyards and apple orchards are sprayed over a longer period from March/April until harvest in September/October. For grassland, forests, water bodies and settlements, we assume that pesticides are applied on spots only.

### 2.2 Passive air sampling

Passive air sampling was conducted in accordance with protocols such as the Global Atmospheric Passive Sampling (GAPS) network (Schuster et al., 2021). We used passive air samplers consisting of two matrices per location: a polyurethane foam (PUF) and a polyester foam (PEF) sampler. Both matrices were placed at about 1.8 m height to simulate a height most related to the inhalation route in humans. The mean distance between samplers was 99.3 ± 49.0 km (mean ± SD) with a minimum distance between samplers of 5.0 km and a maximum distance of 196.8 km. The PUF sampler consisted of a disk (diameter 14 cm, height 1 cm) under a metal dome to avoid direct precipitation of dust and rain. The PEF sampler consisted of four disks (each diameter 8 cm, height 2 cm) placed under the dome and exposed to the air, also collecting particles. PUF disks were obtained from Tisch Environmental Inc. (Cleves, OH, USA), PEF disks from Freudenberg Filtration Technologies (Weinheim, Germany). The combined sampler consisting of PUF and PEF disks was built by TIEM Integrated Environmental Monitoring (Dortmund, Germany) and used to detect both volatile and particle-bound substances such as glyphosate (Kruse-Plaß et al., 2021). In contrast to the German study (Kruse-Plaß et al., 2021) PEF samples were analyzed for the same spectrum of substances as PUF samples. Passive air sampling with PUF matrices has been used in many studies (Gouin et al., 2008; Hayward et al., 2010; Herkert et al., 2018; Koblizkova et al., 2012; Zhang et al., 2013).

Prior to sampling, PUF media were purified using acetone, petroleum ether and methanol (Shoeib et al., 2008). Non-exposed samples were analyzed for both PUF and PEF matrices to account for possible contamination.

Fifteen PUF and six PEF matrices were installed for up to eight months (March to November 2020), six PUF matrices were replaced approximately every two months during this period to account for seasonal variations in pesticide contamination (Supplementary Table S1). Matrices were changed by trained members of the research team using nitrile gloves and forceps according to clear instructions, stored in a cooler, and then placed in a freezer (−18 °C) until laboratory analysis.

### 2.3 Chemical analyses

Chemical analysis of the matrices was performed by the laboratory KWALIS (Fulda, Germany) registered with the German Accreditation Body (Deutsche Akkreditierungsstelle). Samples were analyzed for 566 chemical substances based on the active ingredients listed for plant-based foods in the official multi-method of the German Federal Office of Consumer Protection and Food Safety (ASU L 00.00–115, 2018–10; BVL (2018). Chemical substances to be analyzed included pesticides, their metabolites, safeners, synergists, auxiliary materials, and compounds unrelated to pesticides that are known to exert adverse health effects and may be unintentionally present in agricultural products, such as polychlorinated biphenyls (PCB′s), hexachlorobenzene (HCB), anthraquinone, dichlorobenzophenone (DCBP-pp), and piperonyl butoxide (Kruse-Plaß et al., 2021).

Calculations and statistical analyses for the current study only focused on pesticide active ingredients and their metabolites. Of the 566 substances analyzed, 192 were insecticides (34%), 141 herbicides (25%), and 116 fungicides (20%), 65 substances were metabolites (24%), and 52 other substances (nematicides, rodenticides, acaricides, bactericides, molluscicides, growth regulators, inhibitors, or safeners). This analysis tailored for Germany included 43% of insecticides, 63% of herbicides, and 63% of fungicides approved in Austria during the study year.

Following the protocol DIN EN 15662 (July 2018) chemical substances were analyzed by gas chromatography with mass spectrometry coupling (GC–MS) and/or liquid chromatography-tandem mass spectrometry (LC-MS/MS) in PEF after acetonitrile extraction/partitioning and purification with dispersive SPE sample preparation (QuEChERS) (BVL, 2018). Extraction of PUF was performed with dichloromethane in a Soxhlet extractor (Estellano et al., 2015; Yusà et al., 2009) and analyzed accordingly. In a separate analysis by LC-MS/MS after extraction with aqueous hydrochloric acid and derivatization with fluorenylmethoxycarbonyl, the PEF matrix was analyzed for glyphosate and its primary degradation product aminomethylphosphonic acid (AMPA).

The limit of quantification (LQ) was 10 ng sample^−1^ for most substances. Exceptions were a LQ of 20 ng sample^−1^ for folpet, boscalid (PEF), dimoxystrobin, dodine, fluopyram (PEF), fluxapyroxad (PEF), imidacloprid, prosulfocarb (PEF), tebuconazole (PEF) and thiacloprid. A LQ of 30 ng sample^−1^ was used for chlorothalonil, methylene cyclopropane acetic acid (MCPA), mecoprop-P, desmedipham, pendimethalin (PEF), phenmedipham, propamocarb and prothioconazole-desthio (PEF). For chlorotoluron the LQ was 100 ng sample^−1^, and for glyphosate and AMPA 5 ng sample^−1^.

### 2.4 Meteorological data

The meteorological data are based on the INCA dataset (Haiden et al., 2011) of the Austrian weather service ZAMG. INCA provides gridded meteorological data with a temporal resolution of 1 h for the whole of Austria and a spatial resolution of 1 × 1 km. For the 15 locations, the representative INCA grid was selected and the mean was calculated for the vegetation period (10 March to 20 November) of 2020. For 6 locations, 5 additional subperiods within the vegetation period were calculated. Period averages were calculated for temperature, wind-speed, relative humidity, and radiation, and totals were calculated for precipitation.

### 2.5 Ecotoxicological assessment

All pesticides or their metabolites were characterized with respect to potential harmfulness to the environment and humans according to lists of the Pesticides Properties Database (Lewis et al., 2016), the Pesticide Action Network International (PAN, 2016), or the Austrian Federal Office for Food Safety (BAES, 2021) (Supplementary Table S2). Information on pesticide-specific human toxicity was assessed only for pesticides, as no sufficient information was available for metabolites.

We converted the concentrations sample^−1^ into concentrations m^−2^ assuming that the area of PUF and PEF matrices exposed to air was equal to the land area where the pesticides would be deposited. A total of 311 cm^2^ of one PUF matrix and 205 cm^2^ of four combined PEF matrices were exposed to ambient air.

In order to assess potential toxic loads of detected substances to organisms and ecosystems, we followed common approaches (DiBartolomeis et al., 2019; Goulson et al., 2018; Kudsk et al., 2018; Schulz et al., 2021; Tassin de Montaigu and Goulson, 2020) and divided the concentrations m^−2^ by the reported toxicity/lethality effects for each class of organisms: mammals (acute oral LD_50_ in mg kg^−1^ for rats), birds (acute LD_50_ mg kg^−1^ for different bird spp.), fish (acute 96 h LC50 mg l^−1^ different fish spp.), bees (contact acute LD_50_ μg bee^−1^ for *Apis mellifera*) and earthworms (chronic no observable effect concentration, NOEC, on reproduction of *Eisenia fetida* in mg kg^−1^).

Our toxic load calculations are based upon LD_50_, LC_50_ and NOEC data for each substance from the Pesticide Properties Database accessed in 2021 (Lewis et al., 2016). Because the LD_50_ captures only mortality, our approach does not evaluate sublethal effects or indirect effects, such as dietary depletion. When the reported available LD_50_ values exceeded a certain value (e.g., >2000 mg kg^−1^ of body weight), we used the minimum value (here, 2000 mg kg^−1^). This may overestimate the toxicity of substances with very low toxicity, but we consider this a negligible effect on on the overall calculations.

The calculation of toxic loads is based on the number of LD_50_ doses (in their respective units) of each substance in ambient air. This approach assumes that the substance was absorbed in its entirety by the organism. In reality, however, only a small percentage of the pesticides used will interact with non-target species, perhaps none at all. We do not know the proportion of each compound exposed to non-target organisms; therefore, it is important to reiterate that this is not an attempt to estimate actual death of the non-target organisms under consideration. We also note that the effects of pesticides on organisms can often be indirect (Brühl and Zaller, 2021), e.g., by depleting their food supply, and that sublethal effects can occur at much lower doses than the LD_50_, which we also do not examine here. Nonetheless, we believe this is a useful approach to highlight pesticides that may be worth investigating further in terms of their potential effects.

Human toxicological hazards of detected pesticides was based on the interpretation given in the Pesticide Properties Database (Lewis et al., 2016) and the EU pesticide database (EC, 2021a). Categories distinguished were a substances’ cancerogeneity, reproduction toxicity, endocrine disruption (EDC), acute toxicity, specific target organ toxicity STOT RE/SE (repeated/single exposure), skin irritation, skin sensitization, eye irritation. It is important to note that the interpretations of these databases are a summary of the main human health concerns across a number of issues. However, both use a ‘weight-of-the-evidence’ approach that emphasizes caution (Lewis et al., 2016).

### 2.6 Statistical analyses

All statistical analysis were performed in R (R Core Team 2020). In a first step, the meteorological and land-use parameters (separately) were reduced using principal component analysis (PCA, function in R: princomp) to avoid potential multicollinearity issues. The principal components were then further used as predictor variables in the respective model selection approaches. This was done for each of the data-sets used, i.e. PUF, PEF and time resolved PUF sampling. Only substances > LQ were included in the statistical analysis.

To test the effects of chemo-physical properties, meteorological, and land-use effects on the concentration of individual pesticides, a model selection approach was performed with pesticide concentration (ng sample^−1^) as dependent variable and half-life (DT_50_), bio-concentration factor (1 kg^−1^), volatility (mPa), land-use and meteorological components, and specific pesticide chemical (later referred to as “pesticide”), pesticide class, and interaction between pesticide and pesticide class with all other parameters as predictor variables. Sampling location was included in the model as a random factor. The function buildglmmTMB (buildmer package; Voeten (2020)) was used for automatic AIC-based model selection. After comparing the performance of a number of different distributions (Tweedie, Gaussian – with/without loglink - and negative binomial), the Tweedie distribution provided the best fit (lowest AIC). All model fits performed were visually checked using QQ plots generated with the DHARMa package (Hartig, 2019). Model predictions for the selected models were performed using the ggpredict function in the ggeffects package (Lüdecke, 2018), and ANOVA tables were generated using Anova. glmmTMB. This approach was performed separately for both the PUF and PEF matrices.

To test the effects of the meteorological and land-use variables on total pesticide numbers and concentrations, we performed model selection procedures using either pesticide number per sample or total concentration as dependent variables. The independent variables were the land-use and meteorological components. In this case, a Gaussian error distribution with a logarithmic link (function glm) was used because an initial check showed a good fit. The dredge function (package MuMIn; Bartoń (2020)) was used to compare the AIC of all models, in addition we removed models with a maximum correlation coefficient between predictor variables of rmax > 0.5 in order to avoid issues regarding multi-collinearity (Jaffé (2019) for R code). Model result tables were generated using the function tab_model (package sjPlot (Lüdecke, 2020)). Again, model predictions were estimated using ggpredict. Only data obtained from PUF samplers were used in this analysis.

We tested the hypothesis that increasing number and/or concentration of pesticides leads to increased toxicity by running linear models with total toxic loads (TTL) as the response and number of pesticides or pesticide concentration in the PUF samples as the predictor variable (function glm with a Gaussian error distribution, predictions as above). This was done using data of calculated ecotoxicological parameters for mammals (acute), birds (acute), fish (acute), bees (contact) and earthworms (chronic).

To analyze seasonal effects, we used the temporally resolved PUF samples and total pesticide concentration or number as the dependent variable. Similar to the previous analyses, we performed model selection using the buildglmmTMB function (using a Gaussian error distribution with a logarithmic link, after initial distribution comparisons). The full model included the predictor variables land use and meteorological components, and cosine and sine of time of the year in radians. Sampling location was included in the model as random factor. Cosine and sine of time of the year provide a way to examine the inherently cyclical time variable in linear models (Pewsey et al., 2013). To facilitate interpretation of the results, we present sine(time of the year) and cosine(time of the year) as Sept <> March and Dec <> June, respectively. This represents the minimum (left) and maximum (right) of the cyclic behavior of these variables. Thus, a positive effect of “Sept <> March” would mean higher predicted values in March. Predictions were calculated using ggpredict, and tables were generated using tab_model.

We also plotted the human toxicity of the detected pesticides using the radarchart function in the package fmsb (Nakazawa, 2021).

## 3 Results

### 3.1 Pesticides detected

In total, we found 67 pesticides and 4 metabolites in air sampler matrices which is 27% of the 247 active ingredients approved in Austria in the study year (Table 1). Of the pesticides detected, 45% were fungicides, 36% herbicides, and 19% insecticides. The sampling locations varied considerably in total number of pesticides detected from 10 to 53. In a National Park surrounded by 100% forest 10 pesticides were detected, in another National Park with mainly grassland in the surrounding 33 pesticides, and in a city center surrounded by 100% settlements 17 pesticides were detected (Supplementary Table S3). Nine substances (13% of 67 pesticides found) were not legally approved in Austria during the study period: carbendazim, chlorfenvinphos, cycloate, O,P’-DDT, P,P’-DDT, dichlorprop, dimethenamid, HCB, gamma-HCH, metolachlor, permethrin, tri-allat (Supplementary Table S4); nine substances were also banned under the Stockholm Convention on Persistent Organic Pollutants (HCB, gamma-HCH, 4 PCBs, 3 DDTs).

Most frequently found were five herbicides (metolachlor, pendimethalin, prosulfocarb and terbuthylazine in all samples; and 2,4-D-ethylhexyl in 93% of samples), two fungicides (HCB in all, and chlorothalonil in 93% of samples), and one insecticide (chlorpyriphos-ethyl – in 93% of samples; Table 1). The metabolite most frequently found in 93% of samples was prothiconazole-desthio, the metabolite of the fungicide prothioconazole.

Rankings based on mean concentrations differed from frequency ranks: the highest mean concentrations were for the herbicide prosulfocarb (725 ± 1218 ng sample^−1^), the fungicide folpet (412 ± 465 ng sample^−1^), the insecticide chlorpyrifos-ethyl (110 ± 98 ng sample^−1^) and the metabolite prothioconazole-desthio (75 ± 125 ng sample^−1^) (Table 2).

### 3.2 Analysis of physico-chemical parameters

Predicted concentrations derived from statistical models, holding all influencing factors (land use, meteorology) constant, showed (Supplementary Fig. S1) a predominant influence of a few, highly concentrated fungicides (mainly folpet and chlorothalonil in PUF) and herbicides (prosulfocarb and pendimethalin in PUF; prosulfocarb and glyphosate in PEF). Insecticides were detected in consistently low concentrations in both air sampling matrices (Supplementary Fig. S1). Physicochemical properties did not appear to be related to either concentration or occurrence of the measured substance, as such variables were excluded during automated model selection. It can therefore be assumed that their influence, if any, is small (see Supplementary Table S4).

### 3.3 Relations with land use, meteorological parameters

**Fig. 2:**
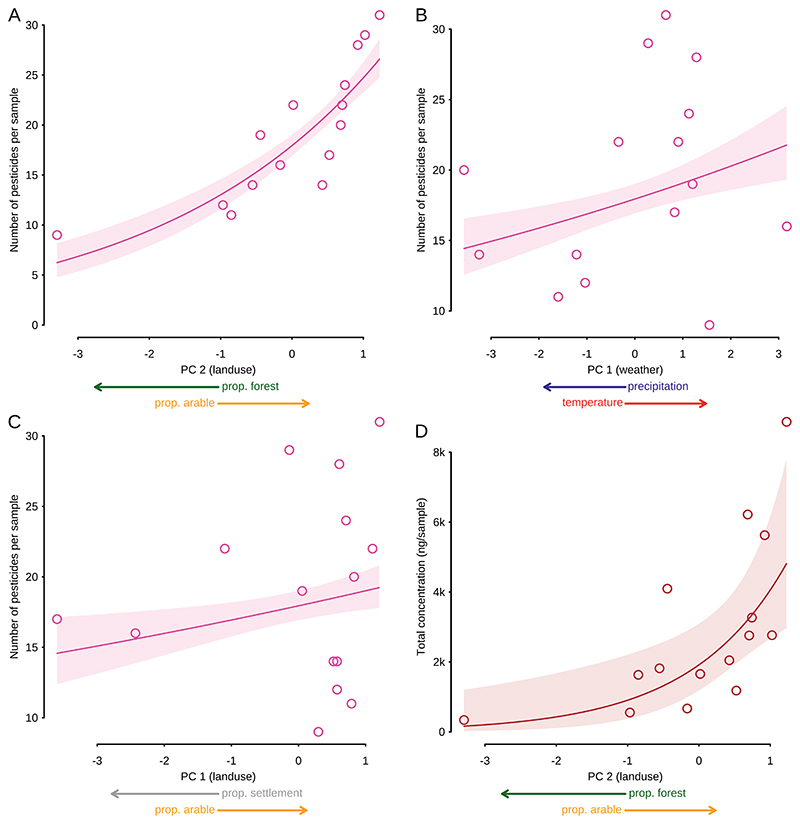
Model predictions of total pesticide numbers (A,B,C) and total concentrations (D) detected with passive air samplers (PUF only) in relation to land use in the surroundings (A,C,D) and meteorological parameters (B). Datapoints show real measurement values (above detection limits), the lines represent the predicted mean, shaded areas 95%-confidence intervals.

After the model selection procedure, the final model for pesticide numbers included the land use axis: settlement vs. arable land; forest vs. arable land; and an axis dominated by arable land and settlement (Fig. 2, Table 3). Thus, arable land indicates the use of pesticides. In addition, a meteorological axis with precipitation and humidity vs. temperature and solar radiation significantly influenced pesticide number. The final model for pesticide concentration included two land-use axes, with only the forest vs. arable land axes showing significance.

Total number of pesticides detected increased steeply with increasing proportion of arable land in the surroundings and decreased with increasing forest area (Fig. 2A; Table 3). Pesticide numbers increased only slightly (but significantly) with increasing arable land vs. settlement area (Fig. 2C, Table 3). Regarding meteorological parameters, the number of pesticides increased with increasing air temperature and decreasing precipitation (Fig. 2B; Table 3).

Total concentrations of pesticides increased steeply with the proportion of arable land and decreasing forest in the surroundings (Fig. 2D; Table 3).

### 3.4 Seasonal fluctuations in pesticide contamination

**Fig. 3:**
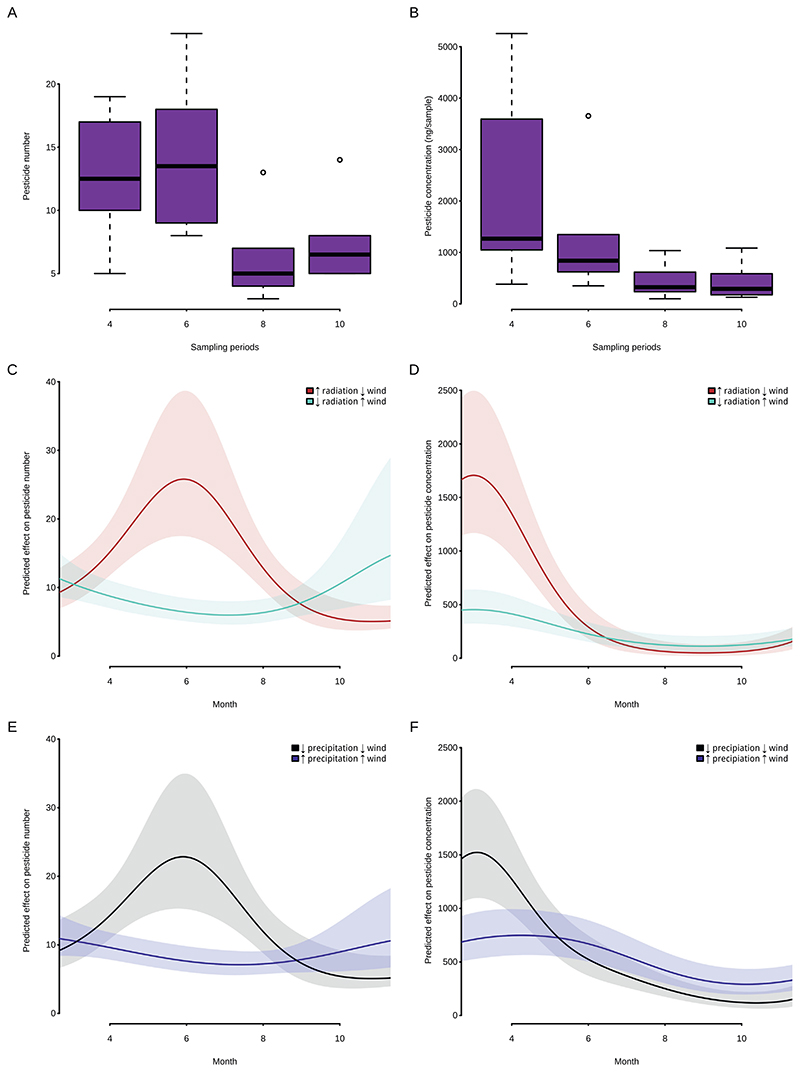
Pesticide numbers (A) and concentrations (B) detected with passive air samplers (PUF only) in different sampling periods. Model predictions for pesticide numbers and concentrations including radiation and wind (C,D) or precipitation and wind (E,F) during the measurement periods. Shades in C—F show 95%-confidence intervals.

Pesticide exposure showed significant seasonal cycles with the highest pesticide numbers in the first half of the year (Fig. 3A) and highest pesticide concentrations in spring (Fig. 3B).

The best models for predicting seasonal variation included similar land use axes as for the analysis over the entire year: proportion of settlement, forest, arable land and vineyards (Supplementary Table S5). As in the previous, the proportion of arable land was found to be positively correlated with pesticide concentration. For pesticide number, this was true only in an interaction with time of the year (Dec <> June). Moreover, seasonal patterns in pesticide numbers and concentrations were clearly time-dependent and interacted with radiation, precipitation and wind speed (Fig. 3C-F). For both, pesticide concentration and number, lower wind combined with higher solar radiation or lower precipitation resulted in the highest predicted values (Fig. 3C-F).

It is important to note that the primary focus of this specific analysis was to understand time-dependent and meteorological patterns. Therefore, time-resolved samples (four samples over the whole period) were used at fewer sites (six locations), so the statistical power to detect land use influences was lower in comparison to the analysis described in the previous subchapter.

### 3.5 Ecotoxicology and human toxicity of detected substances

Ecotoxicological assessments based on LD_50_, LC_50_, or NOEC properties showed significantly increasing toxic loads with increasing pesticide numbers (Supplementary Fig. S2A) and concentrations (Fig. S2A) for most organisms. The exception was the total toxic load for bees, where no significant relationship with pesticide concentration was observed (Fig. S2H).

With the exception of bees, the slopes between toxic loads and concentrations were steeper than those of pesticide numbers.

**Fig. 4:**
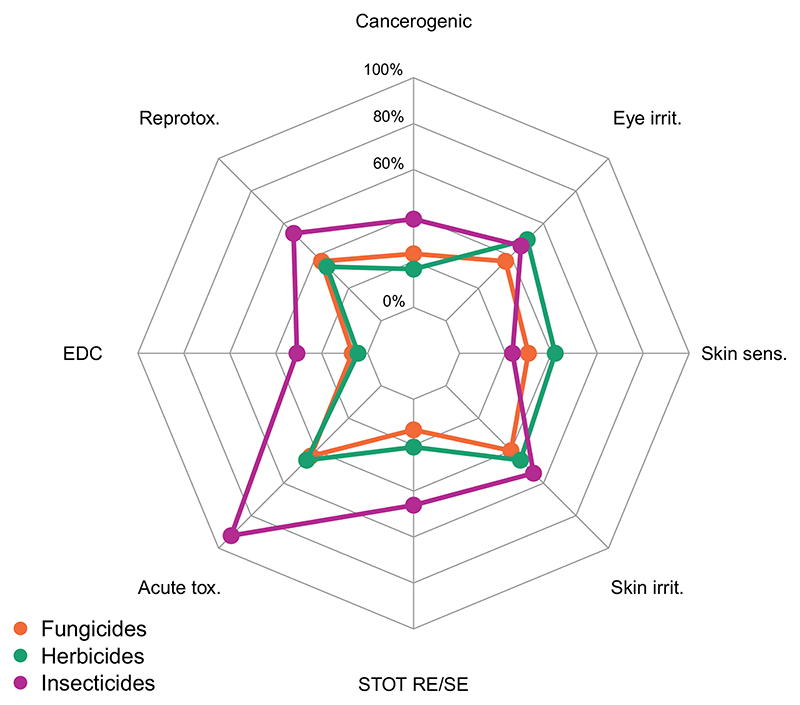
Percentage contribution to human toxicological classifications of fungicides (30 active ingredients), herbicides (24 a. i..), and insecticides (13 a.i.) detected with passive air samplers (PUF only). Only unambiguous classifications were considered. Interpretation of the human health hazards based on the Pesticide Properties Database (Lewis et al., 2016) and EU Pesticide Database (EC, 2021a).

Of the 67 pesticides detected, 36 (53.7%) were classified as acutely toxic to humans, 30 (44.8%) as skin irritants, 29 (43.3%) as eye irritants, 26 (38.8%) as reproductive toxicants, 22 (32.8%) were classified as skin sensitizing, 16 (23.9%) as cancerogenic, 15 (22.4%) as specific target organ toxic, and 7 (10.4%) as endocrine active (Supplementary Table S6 and Table S7, Fig. 4). Four pesticides of the category STOT RE/SE cause respiratory irritation (SE3-hazard code H335): Chlorothalonil, 2,4-D ethylhexyl, cypermethrin, and permethrin.

Detected insecticides had the highest average human toxicity, with 92% of the 13 active ingredients (a.i.) classified as acutely toxic, 54% as reproduction toxicants and skin irritants, 46% as specific target organ toxicants and eye irritants, 38% as cancerogenic, 31% as endocrine disruptors (EDCs), and 23% as skin sensitizers (Fig. 4).

Detected herbicides ranked second in average human toxicity, with 50% of the 24 a.i. classified as eye irritant, 46% as acute toxic and skin irritant, 42% as skin sensitizing, 33% as reproductive toxic, 21% as specific target toxic, 17% as cancerogenic, and 4% as EDCs.

Detected fungicides were the least toxic to humans: 43% of the 30 a.i. classified as acutely toxic, 40% as skin irritants, 37% as eye irritants and reproductive toxicants, 30% as skin sensitizers, 23% as cancerogenic, and 7% as EDCs.

## 4 Discussion

We found that the numbers and concentrations of pesticides (and some metabolites) in the ambient air of a cultural landscape in Austria depend strongly on the surrounding agricultural use. Analysis of seasonal patterns showed a peak in pesticide numbers and concentrations in spring which reflected the main pesticide application activities in the surroundings agricultural fields. Pesticide numbers and concentrations also showed a positive relationship with temperature and radiation, but a negative relationship with precipitation and relative humidity. Contrary to our expectations, the inherent chemical volatility of pesticides did not affect pesticide numbers or concentrations (i.e., was excluded during the model selection procedure). Ambient air contamination with multiple pesticides was not a problem limited to agricultural lands. We found 10 pesticides in samples from a National Park surrounded with 100% forest, 33 pesticides in another National Park with 73% grassland surrounding, and 17 pesticides in samples of a city center surrounded by 100% settlements.

### 4.1 Factors affecting pesticides in ambient air

Of the 67 pesticides in the air samples, 90% were approved for use in arable farming, 82% in horticulture, 46% in fruit orchards, 45% in ornamentals, 37% in vineyards, 19% for private use in home and small gardens, 9% in forestry, and 6% in grassland in Austria (BAES, 2021). Hence, the main source of pesticides in air samples was most likely conventional agriculture. This can be concluded because (i) the majority of detected pesticides have been approved for use in conventional agriculture (but for some the approval expired), (ii) we found a strong positive correlation between agricultural land use and pesticide levels and a negative correlation with forest area. Our analyses also showed that these patterns were driven by a few highly concentrated herbicides and fungicides; insecticides were found in much lower concentrations. Therefore, we assume that either drift during application, or secondary drift after application via volatilization and dust from soil were responsible for pesticide levels in air samples (Deziel et al., 2015; Ward et al., 2006).

Surprisingly, we found 9 pesticides that were not approved in Austria during the sampling period. Some of these pesticides have been banned for decades (e.g., cycloate, DDT, HCB, permethrin) and their origin is therefore unclear. It could be that (i) applicators illegally imported pesticides or depleted their stocks (Zaller, 2020), (ii) officially not approved pesticides were used based on temporary emergency authorizations (mainly involving neonicotinoids), (iii) samples include pesticides with very long half-lives that could be detected decades after application, (iv) contamination from stocks stored at farms (Veludo et al., 2022), and (v) ambient air was contaminated by non-agricultural sources (Linhart et al., 2021). Because only 19% of the detected pesticides were approved for non-professional users, we concluded that the majority of residues in our samples came from agricultural applications. None of the detected pesticides were approved in organic farming. There is also the possibility that pesticides not approved in Austria entered the study areas from neighboring countries, as the distance to the national border of these countries was only between 0.5 and 4 km, depending on the sample location.

A relationship between pesticides in the air and their agricultural use in the surrounding area has also been shown for peach in Israel (Zivan et al., 2017), apple and vine orchards in Northern Italy (Linhart et al., 2019; Linhart et al., 2021) or a mixed agricultural landscape in the Tuscany region in Italy (Estellano et al., 2015). The frequency of pesticide classes found in our air samples most likely depended on their use in surrounding agriculture. Fungicides and herbicides are the dominant pesticide classes in arable farming in Austria (AGES, 2020) and these were also the most frequently found substance classes in our air samples. In landscape more dominated by apple orchards and viticulture fungicides were more dominant in the surroundings (Linhart et al., 2019; Linhart et al., 2021).

### 4.2 Properties of detected pesticides

Five herbicides were detected in all samples; one herbicide, two fungicides, an insecticide, and the metabolite of a fungicide in 93% of samples. This indicates that chemical (adjuvants), technical (noozles to regulate droplet sizes), and regulatory (no-spray restrictions at high wind speeds) measures to prevent pesticide drift (Katzman et al., 2021; Mesnage, 2021) were apparently not sufficient to prevent air contamination in our study region.

We also found glyphosate, the most commonly used herbicide worldwide, in all samples of the PEF matrix at mean concentrations of 116 ± 90 ng sample^−1^. This is comparable to a study using similar air samplers where glyphosate was the only among 500 analyzed substances that was found in all locations across Germany (Kruse-Plaß et al., 2021). In general, there were large differences in the pesticides detected in the current study compared to the German study (Kruse-Plaß et al., 2021), indicating substantial differences in air pollution from pesticides even in similarly structured landscapes. Analyzing the PEF samples for the same range of pesticides as the PUF samples increased the number of detected substances per site. Thus, such a combined analysis allowed the detection of pesticide occurrence comparable to active sampling data (Kruse-Plaß et al., 2021).

The neonicotinoid insecticide in our sample, imidacloprid, had a grace period until 2022, while other neonicotinoids have been banned for outdoor use in the EU since 2018 (EC, 2021b). Neonicotinoids have been linked to detrimentally affect insects (Simon-Delso et al., 2015; van der Sluijs, 2020), soil organisms (van Hoesel et al., 2017), and were found in organically farmed soils and crops (Humann-Guilleminot et al., 2019) and in carnivorous and insectivorous birds (Humann-Guilleminot et al., 2021). Emergency authorizations in Austria (and other EU member states) still allowed their use for short periods in justifiable cases.

Overall, we were surprised to find so many pesticides in ambient air, as only 7% of the 67 pesticides were classified as highly volatile. In addition, the most commonly found herbicides (metolachlor, pendimethalin, prosulfocarb, terbuthylazine and 2,4-D-ethylhexyl), fungicides (HCB, chlorothalonile), and insecticides (chlorpyrifos-ethyl) are all classified as low volatile. This is noteworthy because the risk assessment of pesticides in Europe is also partly based on the volatility of a substance (EFSA, 2014). Thus, our data show that these models seem to underestimate the real situation for non-target organisms, users, bystanders, and neighbors (Clausing, 2020b).

Drift of the herbicides pendimethalin and prosulfocarb causes problems when they contaminate neighboring fields, especially when organic products contaminated with these pesticides can no longer be marketed (Hofmann and Schlechtriemen, 2017). Additionally, sensitive crops (Centner, 2021a) or nature conservation (Brühl et al., 2021a) areas in the surroundings can be negatively affected by pesticide drift. Although the current study was conducted in Austria, the phenomenon of pesticide pollution is a global one (Zaller, 2020) and it has been estimated that 64% of the world’s agricultural land is at risk of pesticide contamination by more than one active ingredient (Tang et al., 2021).

### 4.3 Toxic load for the environment

Pesticides generally have a strong impact on various non-target organisms and are discussed as drivers of biodiversity decline (Brühl and Zaller, 2019; Habel et al., 2019a; Jactel et al., 2020; Poisson et al., 2021; Wagner, 2020). Therefore, we expressed the toxic load of airborne pesticides to mammals, birds, fish, bees and earthworms based on research by several authors (DiBartolomeis et al., 2019; Goulson et al., 2018; Kudsk et al., 2018; Schulz et al., 2021; Tassin de Montaigu and Goulson, 2020). Overall, this is a coarse approach and provides an indication of the potential toxic exposure, but does not imply that a specific number of animals will be killed. Clearly, field-accurate, spatially referenced use data paired with biodiversity monitoring would allow for more accurate risk assessments (Mesnage et al., 2021).

Our models showed that the toxic load for all groups of organisms considered (with the exception of pesticide concentration and bees) increased significantly with increasing number of pesticides and concentration. This is a non-trivial result, as pesticides vary greatly in toxicity and a few very toxic substances can be more toxic than a high number of harmless substances (Schulz et al., 2021; Cech et al., 2022).

Non-target effects of pesticides have been reported for groups of organisms as diverse as plants (Schmitz et al., 2013), soil biota (Gaupp-Berghausen et al., 2015; Gunstone et al., 2021; van Hoesel et al., 2017; Zaller et al., 2021), bees (Sanchez-Bayo and Goka, 2014; Sánchez-Bayo et al., 2016; Sánchez-Bayo and Tennekes, 2020), amphibians (Adams et al., 2020; Baier et al., 2016; Brühl et al., 2013; Leeb et al., 2020a), bats (Stahlschmidt and Brühl, 2012; Stahlschmidt et al., 2017), butterflies at all life stages (Krishnan et al., 2021), and other organisms important for the functioning of agroecosystems (Zaller and Brühl, 2019). In addition to direct toxic effects, amphibians showed avoidance behavior to fungicides and herbicides (Leeb et al., 2020b). Spray drift of herbicides and other pesticides had a significantly negative effect on wildflowers (Brühl and Zaller, 2021; Hahn et al., 2015; Strandberg et al., 2021) that affect floral resources for insect pollinators (Habel et al., 2019b; Kratschmer et al., 2021).

We were surprised to find agricultural pesticides in two National Parks and a city center as well. This is concerning because National Parks are supposed to protect biodiversity and urban biodiversity is already stressed by air pollution, noise and light. Pesticide contamination has also been found in other pristine environments such ice cores of alpine glaciers in Europe (chlorpyrifos; Rizzi et al. (2019), in the Arctic (Balmer et al., 2019) and in insects collected from nature conservation areas in Germany (Brühl et al., 2021a). Using passive air samplers similar to this study, pesticides (endosulfan, cypermethrin, chlorpyrifos) have even been detected in high altitude national parks at >2200 m altitude in Brazil (Guida et al., 2018) or in the Bolivian Andes at altitudes up to 5200 m (Estellano et al., 2008). Potential impacts of these usually low contaminations are poorly understood, but it can be expected that organisms in these ecosystems might be especially sensitive to additional exposures. Although, the amounts found appear negligible, it is important to emphasize that environmental risk assessments are conducted primarily for single substances and that interactions among multiple pesticides, such as those found in the current study, remain unclear.

### 4.4 Potential human health impacts

It was worrying to see that half of the detected pesticides (36 out of 67) were classified as acutely toxic, 39% with reproductive toxicity, 24% as cancerogenic, and 10% as endocrine active. The risks of pesticide exposure of humans especially in agricultural regions, have been documented from various regions including the USA (Lu et al., 2000), Costa Rica (van Wendel de Joode et al., 2012), Italy (Linhart et al., 2021), India (Gupta, 2004), and South Africa (Chetty-Mhlanga et al., 2021).

Quantifying the hazard posed by airborne pesticides is difficult. Our approach was to evaluate the detected pesticides based on the hazard statements provided by the manufacturers but it is difficult to extrapolate those data to realistic scenarios of pesticide exposure of humans living in the study region. However, the point we wanted to make is that humans in the study regions were exposed to substances with different human health risks. Such qualitative decisions without thresholds are also made in the approval of active substances in Europe (Clausing, 2020a). As soon as a substance is classified as cancerogenic, genotoxic or toxic to reproduction no approval may be granted and a quantitative risk assessment is not necessary (Clausing et al., 2018). Of particular concern for airborne contamination are pesticides, which are known to irritate the respiratory tract (STOT RE/SE - hazard code H335). Of these, we found the fungicide chlorothalonil and the herbicide 2,4-D ethylhexyl in almost all samples (93%) and, less frequently, the insecticides permethrin (13%) and cypermethrin (7%). Inhaled pesticides can act differently in the human body when ingested via contaminated food (Clausing, 2020b). However, toxic assessment via ambient air is insufficiently considered during pesticide approval (Clausing, 2020a) and it is argued that the registration process for pesticides is generally a rather outdated assessment system that undervalues health effects (Centner, 2021b).

We also found 9 pesticides listed in the Stockholm Convention of Persistent Organic Pollutants (UNEP, 2019). Persistent organochlorinated pesticides such as the fungicide HCB, which was found in 100% of our samples, pose a public health problem due to their lipophilic properties, which cause bioaccumulation and biomagnification (Mrema et al., 2013).

The pesticides with the highest average concentrations across sample sites were the herbicide prosulfocarb (725 ± 1218 ng sample^−1^), the fungicide folpet (412 ± 465 ng sample^−1^) and the insecticide chlorpyrifos-ethyl (110 ± 98 ng sample^−1^). Prosulfocarb is contained in 7 products approved in Austria and widely used in arable farming and horticulture; folpet is contained in 27 fungicides applied in vineyards, cereals and horticulture. Chlorpyrifos-ethyl, was widely used in arable farming, fruit and wine orchards and horticulture but has been banned in EU since 2020.

Although the amounts found in ambient air were low, it is of concern that 7 (10%) of the pesticides (dimoxystrobin, HCB, bromoxynil, chlorfenvinphos, permethrin, O,P′-DDT, thiacloprid) were endocrine active or endocrine disrupting chemicals (EDCs), and 25 pesticides are possibly EDCs.These substances affect hormone balance in fetuses, children and adolescents (Kahn et al., 2020) and can be effective at very low doses (Mesnage and Antoniou, 2021). Importantly, even very low levels of pesticide exposure over years can affect children’s development, health, and behavior (Pascale and Laborde, 2020). Simultaneous exposure of a variety of pesticides could also trigger synergistic effects (Laetz et al., 2009), which are extremely difficult to study in risk assessments (Damalas and Eleftherohorinos, 2011). Because of the low-dose effect, the non-monotonic dose-response relationship and the interaction of EDCs with endogenous hormones or other EDCs, it is questionable whether a low dose can ever be considered safe (Munn and Goumenou, 2013; Vandenberg et al., 2012).

Among the pesticides detected, the insecticides chlorpyrifos-ethyl and chlorpyrifos-methyl were among the most hazardous. Since 2020, these two insecticides have been banned in the EU and airborne contamination should be reduced in the future. Residues of these chemicals have also been found on children’s playgrounds near intensively managed apple orchards (Linhart et al., 2019) and other public lands (Linhart et al., 2021), and in urine samples of agricultural workers and residents living in agricultural areas (Paglia et al., 2021). Even very low doses of chlorpyr-ifos have been shown to cause brain abnormalities in fetuses and children (Rauh et al., 2012), and impair locomotor activity, behavior, and neurotransmitter systems in rats (Perez-Fernandez et al., 2020).

All the sites we studied had multiple contamination with pesticides, but the effects of multiple exposures are rarely studied. Such studies would be necessary since it is obvious that people living in such areas are simultaneously exposed by breathing the contaminated air.

## 5 Conclusions

We detected multiple pesticides in the air in an Austrian cultural landscape not only in agricultural areas, but also in two National Parks and even inmidst of a large city. Several of these pesticides potentially pose a toxicological risk to organisms, including humans, but the effects of such contamination are not well known. One aspect that is rarely addressed in scientific and public debate is that pesticides found in air samples may also be deposited in private gardens, nature reserves, or organic farm fields.

Our findings also suggest that regulatory guidance on operator, worker, resident, and bystander exposure (EFSA, 2014) is inadequate. Therefore, we recommend a general reduction of pesticide exposure as foreseen in the European form-to-fork strategy for sustainable agriculture (Brühl et al., 2021b; EC, 2019). A prerequisite for further research would be systematic monitoring of pesticides in air and other media, as already exists for pesticide monitoring of food (EPRS, 2018). Overall, our results show that pesticide application practices should be improved as precautionary measures to protect human and environmental health from uncontrolled pesticide exposure.

## Supplementary Material

Figure S1

Figure S2

Supplementary tables

## Figures and Tables

**Table 1:** Overview of number of pesticides detected with passive air sampler using polyurethan foam (PUF) and polyethylene foam (PEF) matrices. Residues above and below detection limit are listed.

Parameter/	Sampling method	
Number of…	PUF	PEF
Samples	15	6
Detected substances	56	38
Detected substances site^−1^ (median)	10–35 (23)	7–35 (16)
Substances detected only in this matrix	39	20
Metabolites	2	3
Pesticides / metabolites detected in total	67 / 4	
Fungicides / herbicides / insecticides	30 / 24 / 13	
Volatility low (<5 mPa) / moderate / high (>10 mPa)^a^	58/1/5	
Not approved substances in 2020	9	
Substances banned under the Stockholm convention^b^	9	

a Without categorization: chlorotoluron, O,P′ -DDT und P,P′ -DDT.

b Stockholm Convention on persistent organic pollutants (UNEP, 2019).

**Table 2:** Top five most frequently found substances (only PUF matrix considered). Min values refer to lowest concentrations detected, when the substance was present. See Supplementary Table S4 for all detected substances.

Substance	Frequency found in	Rank based on	Concentration (ng sample^−1^)			Rank based on
	% samples	frequency	min	max	Mean ± SD	mean conc.
Herbicides						
Metolachlor	100	1	12.3	382.6	117.2 ± 98.1	3
Pendimethalin	100	1	44.9	3932.4	650.4 ± 1044.9	2
Prosulfocarb	100	1	13.7	4758.8	724.7 ± 1217.9	1
Terbuthylazine	100	1	15.5	583.6	95.3 ± 139.3	5
2,4-D-ethylhexyl	93.3	2	12.0	71.0	30.9 ± 23.5	9
Fungicides						
HCB	100	1	14.0	43.4	28.1 ± 8.7	4
Chlorothalonile	93.3	2	30.6	554.4	193.6 ± 177.7	2
Folpet	86.7	3	35.5	1665.2	411.9 ± 465.3	1
Tebuconazole	80.0	4	10.4	67.7	23.6 ± 21.5	5
Tetraconazole	53.3	5	11.1	61.3	17.1 ± 21.0	7
Insecticides						
Chlorpyrifos-ethyl	93.3	1	24.7	287.0	110.3 ± 98.3	1
Chlorpyrifos-methyl	53.3	2	15.5	126.5	23.8 ± 34.4	3
Tefluthrin	33.3	3	18.5	91.8	13.1 ± 25.0	4
Gamma-HCH	26.7	4	10.5	27.0	4.0 ± 7.8	5
P,P′-DDT	13.3	5	12.5	15.5	1.9 ± 5.0	7
Permethrin	13.3	5	17.3	37.5	3.7 ± 10.4	6
Metabolites						
Prothioconazole-desthio	93.3	1	14.4	501.0	74.9 ± 124.7	1
P,P′-DDE	73.3	2	11.6	109.5	31.4 ± 34.6	2

**Table 3:** Statistical analysis of total numbers and concentrations of pesticides in passive air samplers (PUF only) in response to land use in the surroundings and meteorological parameters. Significant effects in bold.

Predictors	t	p
Number of pesticides		
Settlement <> arable land^1^	2.68	0.023
Forest <> arable land	8.94	<0.001
Arable land & settlement	−4.05	0.002
Precipitation & humidity <> temperature & radiation	3.51	0.006
Concentrations of pesticides		
Settlement <> arable land	1.85	0.089
Forest <> arable land	2.91	0.013

<> denotes the axis range in the model, i.e. settlement arable land together with a positive t-value means that number of pesticides increases with proportion of arable land.

## Data Availability

The datasets used and/or analyzed during the current study are available from the corresponding author on reasonable request.

## Highlights

- Passive air sampling detected 67 different pesticides.
- These included 24 herbicides, 30 fungicides, and 13 insecticides.
- Pesticides increased with increasing arable land in the surroundings.
- Pesticides also found in two National Parks and a City Center.
- Potential toxicity for non-target organisms and humans was considerable.
