# Supplementary figures and images for: Pesticides in ambient air, influenced by surrounding land use and weather, pose a potential threat to biodiversity and humans
Source: Sci Total Environ. Author manuscript; Available in PMC 2023 Apr 7. (PMC7614392; doi:10.1016/j.scitotenv.2022.156012)

A

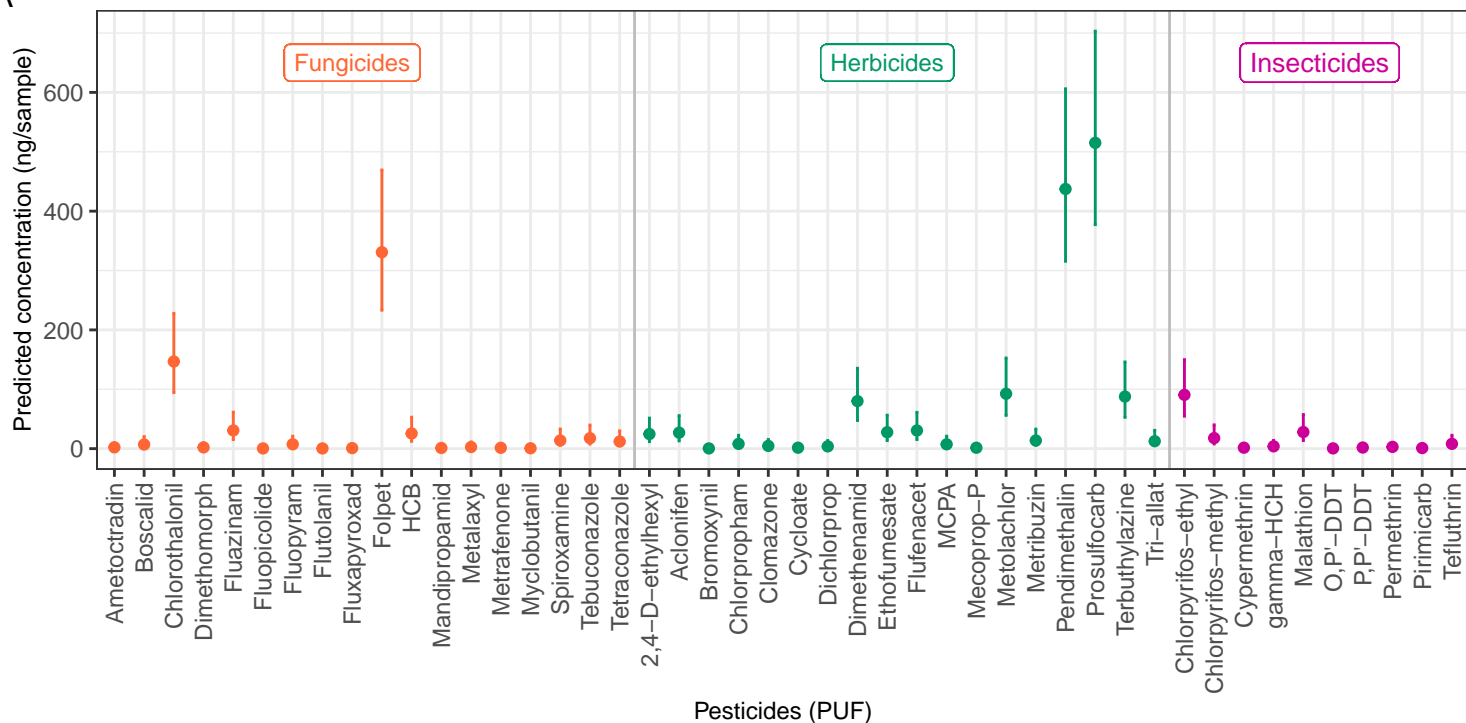

B

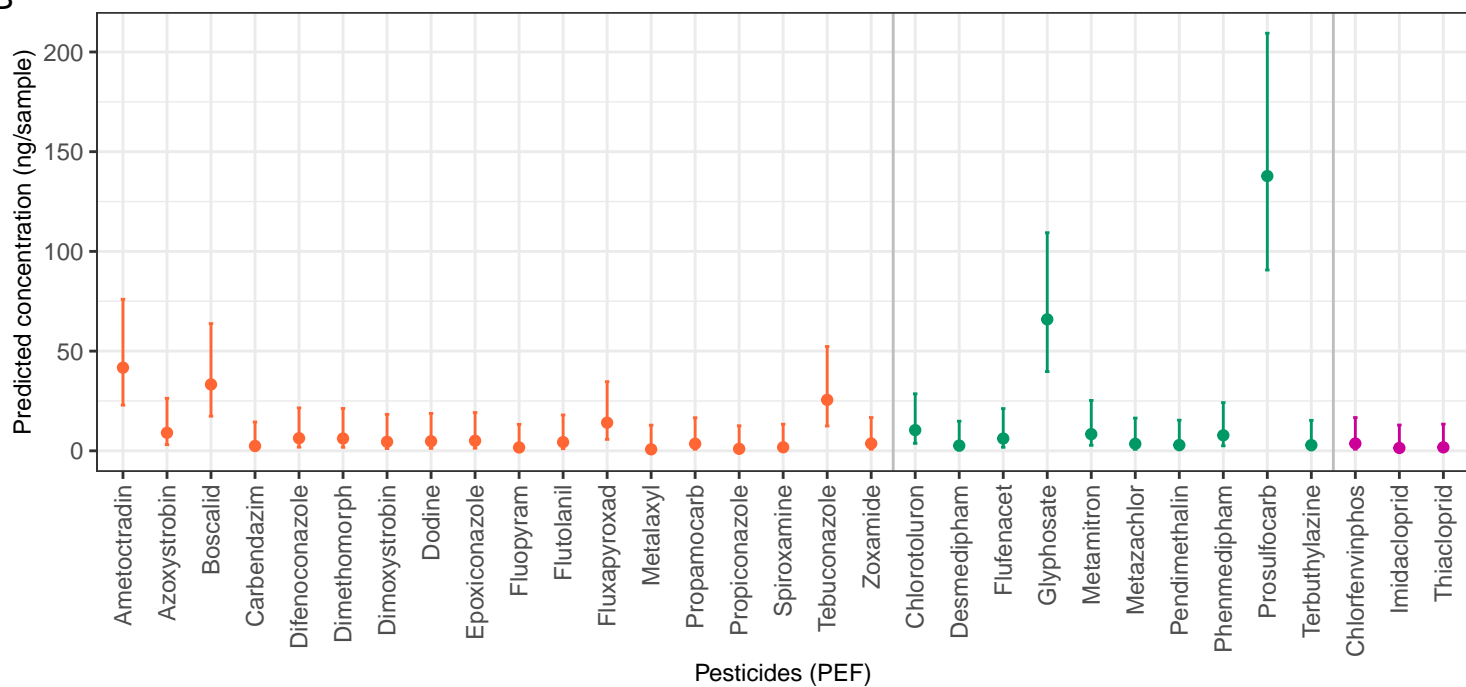

Supplement: Figure S1 [file EMS172737-supplement-Figure_S1.pdf]

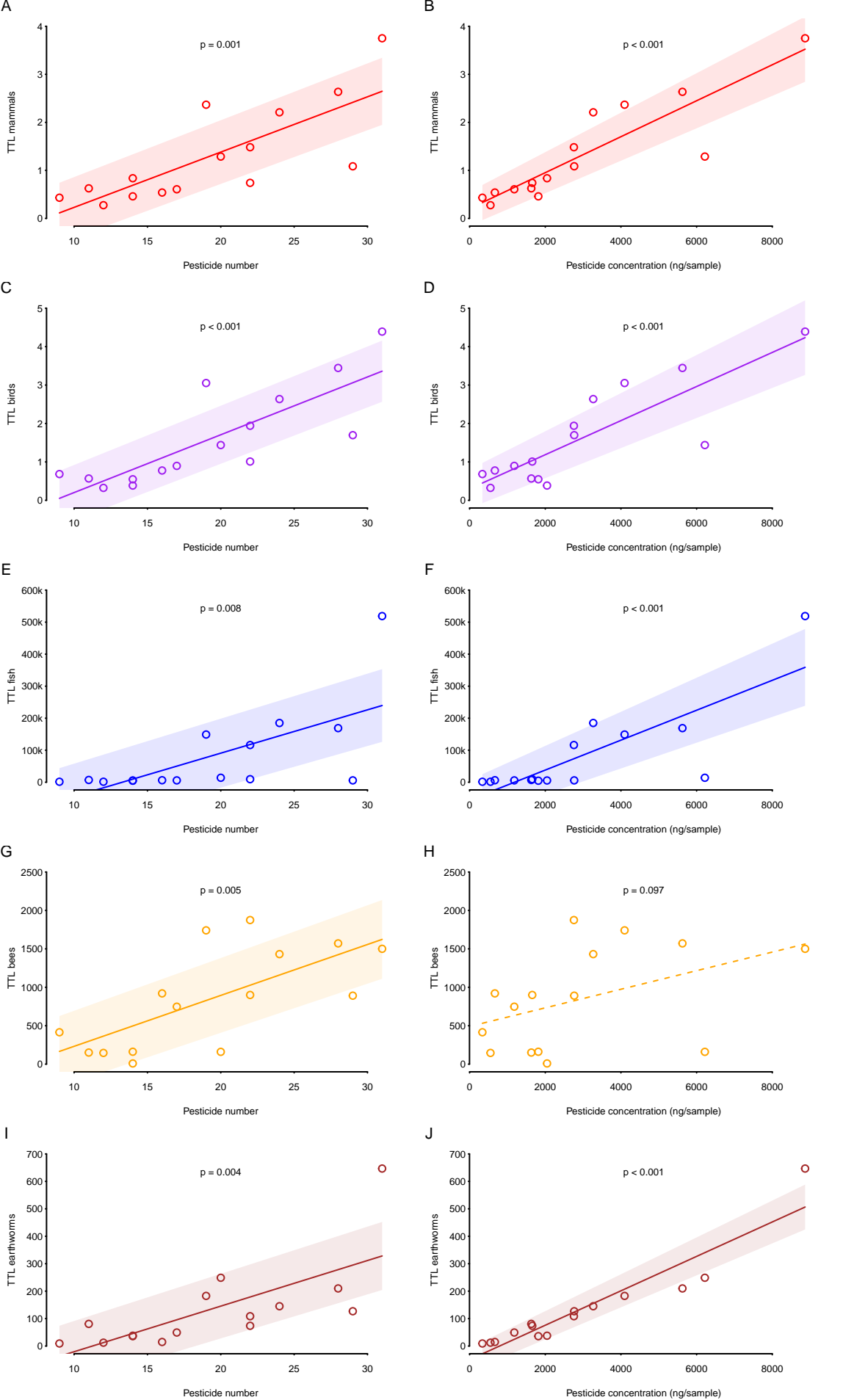

Supplement: Figure S2 [file EMS172737-supplement-Figure_S2.pdf]
