# Supplementary material for: Pesticides in ambient air, influenced by surrounding land use and weather, pose a potential threat to biodiversity and humans
Source: Sci Total Environ. Author manuscript; Available in PMC 2023 Apr 7. (PMC7614392; doi:10.1016/j.scitotenv.2022.156012)
Supplement: Supplementary tables [file EMS172737-supplement-Supplementary_tables.docx]

**Table S1.** Overview of exposition times of the two air sampler matrices polyurethan foam (PUF) and polyethylene foam (PEF). Six of the 15 sites were analysed in time intervals.

| Sample | Number  of sites | Start exposition  year 2020 | End exposition  year 2020 | Duration of exposition  (mean days) |
| --- | --- | --- | --- | --- |
| Polyurethan foam (PUF) | 15 | 02-13 Mar | 17-21 Nov | 257 |
|  | 6 | 02-10 Mar | 18-26 May | 75 |
|  | 6 | 18-26 May | 20-24 Jul | 62 |
|  | 6 | 20-24 Jul | 21-24 Sep | 63 |
|  | 6 | 21-24 Sep | 17-19 Nov | 56 |
| Polyethylen foam (PEF) | 6 | 07-10 Mar | 18-20 Nov | 256 |

**Table S2**. Parameters assessed to characterize physico-chemical parameters, ecotoxicology, human toxicity and approval of analyzed substances. DT_50_…soil half-life, LD_50_…lethal dosis, LC_50_…lethal concentration, NOEC…no observed effect concentration.

| Physico-chemical  parameters^1^ | Overall toxicity^2^  Highly hazardous  pesticide (HHP) | Ecotoxicity^1^  (mg kg^-1^, mg l^-1^ fish,  µg bee^-1^) | Human  toxicity^1^ | Approved for  which sector in Austria^3^ |
| --- | --- | --- | --- | --- |
| Volatility (vapor press.) | Acute toxicity | Mammals acute oral LD_50_ | Eye irritating | Approval yes/no |
| Half-life DT_50_ field | Long-term effects | Birds acute LD_50_ | Skin sensitive | Organic farming |
| Bio-concentration factor | Environm. toxicity | Fish acute 96h LC_50_ | Skin irritating | Arable crops |
|  | Banned under  Stockholm  convention | Bees contact LD_50_  Earthw. NOEC chronic | Specific target organ toxicity | Vegetables |
|  |  |  | Acute toxicity | Grassland |
|  |  |  | Endocrine act. | Fruit orchards |
|  |  |  | Reproduction tox. | Vineyards |
|  |  |  | Cancerogenic | Hops |
|  |  |  |  | Ornamentals |
|  |  |  |  | Forestry |
|  |  |  |  | Post harvest use |
|  |  |  |  | Non agriculture |
|  |  |  |  | Private use |

Sources: ^1^Pesticides Properties Database (Lewis et al., 2016) and EU pesticide Database (ec.europa.eu/food/plant/pesticides/eu-pesticides-database/), ^2^(PAN, 2016), ^3^(BAES, 2021)

**Table S3. Overview of detected pesticide residues in ambient air at different locations in eastern Austria using PUF and PEF passive samplers. All residues above determination limit were counted.**

|  | Number of residues detected… | | |  |
| --- | --- | --- | --- | --- |
| Location | Fungicides | Herbicides | Insecticides | Total |
| 1 | 27 | 18 | 8 | 53 |
| 2 | 18 | 12 | 5 | 35 |
| 3 | 12 | 14 | 1 | 27 |
| 4 | 11 | 13 | 5 | 29 |
| 5 | 9 | 12 | 4 | 25 |
| 6 | 17 | 15 | 3 | 35 |
| 7 | 5 | 10 | 0 | 15 |
| 8 | 2 | 9 | 1 | 12 |
| 9 | 5 | 9 | 1 | 15 |
| 10 | 3 | 9 | 1 | 13 |
| 11 | 11 | 12 | 3 | 26 |
| 12 | 15 | 14 | 4 | 33 |
| 13 | 7 | 7 | 4 | 18 |
| 14 | 2 | 7 | 1 | 10 |
| 15 | 5 | 8 | 4 | 17 |

**Table S4. Pesticide active ingredients detected in passive samplers (PUF only). *…only detected in one sample, min values refer to lowest concentrations detected, if the substance was present.**

| Substance | Found in | | Concentration (ng sample^-1^) | | | |
| --- | --- | --- | --- | --- | --- | --- |
|  | % samples | min | | max | mean | SD |
| *Herbicides* |  |  | |  |  |  |
| Metolachlor | 100 | 12.3 | | 382.6 | 117.2 | 98.1 |
| Pendimethalin | 100 | 44.9 | | 3932.4 | 650.4 | 1044.9 |
| Prosulfocarb | 100 | 13.7 | | 4758.8 | 724.7 | 1217.9 |
| Terbuthylazine | 100 | 15.5 | | 583.6 | 95.3 | 139.3 |
| 2,4-D-ethylhexyl | 93.3 | 12.0 | | 71.0 | 30.9 | 23.5 |
| Tri-allat | 86.7 | 11.2 | | 33.2 | 15.4 | 8.2 |
| Dimethenamid | 80.0 | 14.7 | | 785.5 | 102.0 | 196.3 |
| Ethofumesate | 66.7 | 17.6 | | 160.1 | 37.9 | 46.5 |
| Flufenacet | 60.0 | 10.2 | | 283.3 | 33.0 | 72.0 |
| Aclonifen | 40.0 | 10.8 | | 534.9 | 47.0 | 136.7 |
| Clomazone | 33.3 | 10.0 | | 28.3 | 6.1 | 9.9 |
| Metribuzin | 33.3 | 14.9 | | 123.0 | 21.1 | 37.3 |
| MCPA | 20.0 | 34.5 | | 68.8 | 10.6 | 22.8 |
| Chlorpropham | 13.3 | 43.3 | | 166.3 | 14.0 | 43.6 |
| Cycloate | 13.3 | 10.0 | | 24.5 | 2.3 | 6.7 |
| Dichlorprop | 13.3 | 14.1 | | 61.9 | 5.1 | 16.1 |
| Bromoxynil* | 6.7 | 10.9 | | 10.9 | 0.7 | 2.8 |
| Mecoprop-P* | 6,7 | 33.3 | | 33.3 | 2.2 | 8.6 |
|  |  |  | |  |  |  |
| *Fungicides* |  |  | |  |  |  |
| HCB | 100 | 14.0 | | 43.4 | 28.1 | 8.7 |
| Chlorthalonil | 93.3 | 30.6 | | 554.4 | 193.6 | 177.7 |
| Folpet | 86.7 | 35.5 | | 1665.2 | 411.9 | 465.3 |
| Tebuconazole | 80.0 | 10.4 | | 67.7 | 23.6 | 21.5 |
| Tetraconazole | 53.3 | 11.1 | | 61.3 | 17.1 | 21.0 |
| Boscalid | 46.7 | 15.6 | | 60.0 | 10.5 | 15.9 |
| Fluazinam | 46.7 | 17.2 | | 318.4 | 48.4 | 94.5 |
| Fluopyram | 46.7 | 10.1 | | 54.4 | 10.2 | 15.2 |
| Spiroxamine | 40.0 | 11.6 | | 188.8 | 19.7 | 48.6 |
| Ametoctradin | 20.0 | 10.2 | | 23.0 | 2.9 | 6.6 |
| Dimethomorph | 20.0 | 10.8 | | 23.6 | 3.1 | 7.0 |
| Metalaxyl | 20.0 | 14.1 | | 37.5 | 4.4 | 10.4 |
| Fluopicolide* | 6.7 | 11.3 | | 11.3 | 0.8 | 2.9 |
| Flutolanil* | 6.7 | 10.2 | | 10.2 | 0.7 | 2.6 |
| Fluxapyroxad* | 6.7 | 19.1 | | 19.1 | 1.3 | 4.9 |
| Mandipropamid* | 6.7 | 30.9 | | 30.9 | 2.1 | 8.0 |
| Metrafenone* | 6.7 | 18.4 | | 18.4 | 1.2 | 4.7 |
| Myclobutanil* | 6.7 | 14.1 | | 14.1 | 0.9 | 3.6 |
|  |  |  | |  |  |  |
| *Insecticides* |  |  | |  |  |  |
| Chlorpyrifos-ethyl | 93.3 | 24.7 | | 287.0 | 110.3 | 98.3 |
| Chlorpyrifos-methyl | 53.3 | 15.5 | | 126.5 | 23.8 | 34.4 |
| Tefluthrin | 33.3 | 18.5 | | 91.8 | 13.1 | 25.0 |
| gamma-HCH | 26.7 | 10.5 | | 27.0 | 4.0 | 7.8 |
| P,P’-DDT | 13.3 | 12.5 | | 15.5 | 1.9 | 5.0 |
| Permethrin | 13.3 | 17.3 | | 37.5 | 3.7 | 10.4 |
| Cypermethrin* | 6.7 | 24.5 | | 24.5 | 1.6 | 6.3 |
| O,P’-DDT* | 6.7 | 10.4 | | 10.4 | 0.7 | 2.7 |
| Malathion* | 6.7 | 628.8 | | 628.8 | 41.9 | 162.3 |
| Pirimicarb* | 6.7 | 27.2 | | 27.2 | 1.8 | 7.0 |

**Table S5. Statistical analysis of pesticide numbers and concentrations in passive air samplers (PUF only) in response to sampling period, land use or meteorological parameters. Significant effects in bold.**

| Predictors | t | P |
| --- | --- | --- |
| *Number of pesticides* |  |  |
| Sept <> March^1^ | 3.91 | **<0.001** |
| Settlement & forest <> arable land | 1.15 | 0.249 |
| Dec <> June | -1.84 | 0.065 |
| Wind <> radiation | -0.20 | 0.840 |
| Precipitation & wind | 0.32 | 0.752 |
| Dec <> June by wind <> radiation | -4.15 | **<0.001** |
| Dec <> June by precipitation & wind | 3.04 | **0.002** |
| Settlement & forest <> arable land by Dec <> June | -2.76 | **0.006** |
|  |  |  |
| *Concentrations of pesticides* |  |  |
| Sept <> March | 8.33 | **<0.001** |
| Humidity & temperature | 1.51 | 0.131 |
| Vineyard & settlement <> forest | -1.92 | 0.054 |
| Dec <> June | -7.05 | **<0.001** |
| Settlement & forest <> arable land | 5.54 | **<0.001** |
| Wind <> radiation | -0.87 | 0.382 |
| Grassland <> arable land | 2.10 | **0.036** |
| Sept <> March by vineyard & settlement <> forest | 4.21 | **<0.001** |
| Dec <> June by wind <> radiation | -3.94 | **<0.001** |
| Humidity & temperature by Dec <> June | -3.98 | **<0.001** |
| Sept <> March by grass <> arable land | -4.90 | **<0.001** |
| Dec <> June by grass <> arable land | 3.34 | **0.001** |

^1^ <> denotes the axis range in the model, i.e. Sept <> March together with a positive t-values means that number of pesticides increases towards March and decreases towards September.

**Table S6 Human health characteristics of pesticide substances collected with passive samples. Source: PPDB, *EUpestDB;** sens = sensitization, irrit = irritant, dam = damage, STOT RE/SE = specific target organ toxicity — RE repeated exposure, SE single exposure, ✓ = known to cause a problem, X = known not to cause a problem, ? = possibly, status not identified; n.d. = no data; sum = sum of p,p-DDT, o,p-DDT, p-p-DDE and p,p-TDE.

| Chemical | Cancerogen | Reprotox | EDC | Acute tox* | STOT RE/SE* | Skin irrit. | Skin sens. | Eye irrit. |
| --- | --- | --- | --- | --- | --- | --- | --- | --- |
| **Fungicides** | **7** | **11** | **2** | **13** | **4** | **12** | **9** | **11** |
| Ametoctradin | X | ? | X | na | na | X | n.d. | X |
| Azoxystrobin | X | ? | n.d. | 3 | na | ✓ | n.d. | ✓ |
| Benthiavalicarb isopropyl | ✓ | ? | X | na | na | X | ✓ | X |
| Boscalid | ? | ? | X | na | na | X | X | X |
| Carbendazim | ? | 1B* | ? | na | na | X | n.d. | X |
| Chlorothalonil | 2* | ✓ | ? | 2 | 3SE | ✓ | 1* | ✓/1dam* |
| Difenoconazole | ? | ? | X | na | na | ✓ | n.d. | ✓ |
| Dimethomorph | ✓ | ? | n.d. | na | na | ✓ | X | ✓ |
| Dimoxystrobin | 2* | 2* | ✓ | 4 | na | n.d. | n.d. | n.d. |
| Dodine | X | ? | n.d. | 4 | na | 2* | n.d. | 2* |
| Epoxiconazole | 2* | 1B* | ? | na | na | X | ? | X |
| Fluazinam | ? | 2* | ? | 4 | na | ✓ | 1A* | ✓irrit/1dam* |
| Fluopicolide | X | X | n.d. | na | na | X | n.d. | X |
| Fluopyram | X | ? | n.d. | na | na | X | n.d. | X |
| Flutolanil | X | X | X | na | na | X | X | X |
| Fluxapyroxad | ? | ? | n.d. | na | na | X | n.d. | ? |
| Folpet | 2* | n.d. | n.d. | 4 | na | ✓ | 1* | 2* |
| HCB | 1B* | ? | ✓ | na | 1RE | ✓ | n.d. | X |
| Mandipropamid | X | X | X | na | na | X | ✓ | X |
| Metalaxyl | X | X | X | 4 | na | X | 1* | X |
| Metrafenone | ? | ✓ | n.d. | na | na | ✓ | n.d. | ✓ |
| Myclobutanil | X | 2* | ? | 4 | na | X | n.d. | 2* |
| Oxathiapiprolin | X | ? | n.d. | na | na | X | X | X |
| Propamocarb | n.d. | n.d. | ? | na | na | ✓ | ? | X |
| Propiconazole | ? | 1B* | ? | 4 | na | X | 1* | X |
| Pyraclostrobin | X | ✓ | X | 3 | na | 2* | X | X |
| Spiroxamine | X | 2* | n.d. | 4 | 2RE | 2* | 1* | X |
| Tebuconazole | ? | 2* | n.d. | 4 | 2 | X | n.d. | ✓ |
| Tetraconazole | ? | ? | n.d. | 4 | na | X | n.d. | X |
| Zoxamide | X | X | n.d. | na | na | ? | 1* | ✓ |
|  |  |  |  |  |  |  |  |  |
| **Herbicides** | **4** | **8** | **1** | **11** | **5** | **11** | **10** | **12** |
| 2,4-D-ethylhexyl | ? | ✓ | ? | 4 | 3SE | ? | 1* | ✓irrit/1dam* |
| Aclonifen | 2* | ? | n.d. | na | na | ✓ | 1A* | X |
| Bromoxynil | ? | 2* | ✓ | 2/3 | na | ✓ | 1* | X |
| Chlorpropham | 2* | ? | n.d. | na | 2RE | X | X | X |
| Chlorotoluron | 2* | 2* | ? | na | na | X | n.d. | X |
| Clomazone | X | ✓ | n.d. | na | na | ? | X | ? |
| Cycloate | X | ? | n.d. | na | na | ✓ | ? | ✓ |
| Desmedipham | ? | ✓ | ? | na | na | ? | ? | X |
| Dichlorprop | n.d. | ? | n.d. | 4 | na | 2* | n.d. | ✓irrit/1dam* |
| Dimethenamid | ? | n.d. | n.d. | na | na | ✓ | ✓ | X |
| Ethofumesate | X | ? | n.d. | na | na | X | n.d. | X |
| Flufenacet | X | ? | n.d. | 4 | 2RE | ? * | 1* | ? |
| Glyphosate | ? | X | ? | na | na | ✓ | n.d. | ✓irrit/1dam* |
| MCPA | X | ? | X | 4 | na | 2* | n.d. | ✓irrit/1dam* |
| Mecoprop-P | ? | ✓ | X | 4 | na | X | X | ✓irrit/1dam* |
| Metamitron | X | ? | n.d. | 4 | na | X | n.d. | Xirrit/1dam* |
| Metazachlor | 2* | ? | n.d. | na | na | ✓ | 1B* | ✓ |
| Metolachlor | ? | ? | ? | na | na | ✓ | ? | ✓ |
| Metribuzin | X | ✓ | ? | 4 | na | X | X | X |
| Pendimethalin | ? | ✓ | ? | na | na | ✓ | 1* | ✓ |
| Phenmedipham | ? | ? | n.d. | na | na | X | ? | X |
| Prosulfocarb | n.d. | n.d. | n.d. | 4 | na | ✓ | 1* | ✓ |
| Terbuthylazine | ? | ? | n.d. | 4 | 2RE | ? | ✓ | ✓ |
| Tri-allat | ? | ? | n.d. | 4 | 2RE | ? | 1* | X |
|  |  |  |  |  |  |  |  |  |
| **Insecticides** | **5** | **7** | **4** | **12** | **6** | **7** | **3** | **6** |
| Chlorfenvinphos | n.d. | n.d. | ✓ | 2/3 | na | n.d. | n.d. | n.d. |
| Chlorpyrifos-ethyl | X | ✓ | ? | 3 | na | X | ? | X |
| Chlorpyrifos-methyl | X | n.d. | ? | na | na | ✓ | 1* | X |
| Cypermethrin | ? | ? | ? | 4 | 3SE | X | X | ✓ |
| Gamma-HCH | ? | ? | ? | 3/4 | 2RE | ✓ | n.d. | ✓ |
| Imidacloprid | X | ✓ | n.d. | 4 | na | ? | n.d. | ? |
| Malathion | ? | ? | ? | 4 | na | X | 1* | X |
| Permethrin | ✓ | ✓ | ✓ | 4 | 3SE | ✓ | n.d. | ✓ |
| O,P’-DDT | 2sum* | ✓ | ✓ | 3sum | 1REsum | ✓ | n.d. | n.d. |
| P,P’-DDT | 2sum* | ✓ | ? | 3sum | 1REsum | ✓ | n.d. | ? |
| Pirimicarb | 2* | ✓ | n.d. | 3 | na | ? | 1* | ✓ |
| Tefluthrin | X | ? | ? | 1/2 | na | ✓ | n.d. | ✓ |
| Thiacloprid | 2* | 1B* | ✓ | 3/4 | 3SE | ✓ | X | ✓ |
| **Total effective pesticides** | **16** | **26** | **7** | **36** | **15** | **30** | **22** | **29** |

**Table S7. Overview of human health characteristics of the 67 detected pesticide categories collected with passive air samples. Assessment according to Pesticides Properties Database (Lewis et al., 2016)^1^, EU Pesticides Database (EC, 2021a)^2^. STOT RE/SE - specific target organ toxicity, RE repeated exposure, SE single exposure; a.i. - Only substances with unambiguous assignments are listed; multiple assignments to health characteristics are possible; for details on all detected substances see Supplementary Table S2.**

| Pesticide class | Eye irritant^1^ | Skin sens-itisation^1^ | Skin irritant^1^ | STOT RE/SE^2^ | Acute toxicity^2^ | Endocrine activity (EDC)^1^ | Reprod. toxicity^1^ | Cancero-genity^1^ |
| --- | --- | --- | --- | --- | --- | --- | --- | --- |
| Fungicides (30 a.i.) | 11 | 9 | 12 | 4 | 13 | 2 | 11 | 7 |
| Herbicides (24 a.i.) | 12 | 10 | 11 | 5 | 11 | 1 | 8 | 4 |
| Insecticides (13 a.i.) | 6 | 3 | 7 | 6 | 12 | 4 | 7 | 5 |
| Total | 29 | 22 | 30 | 15 | 36 | 7 | 26 | 16 |
